# Urinary tract infection during pregnancy and time relation to preterm birth—a Swedish observational study

**DOI:** 10.1111/aogs.70156

**Published:** 2026-02-13

**Authors:** Karin Dahlquist, Andrea Stuart, Karin Källén

**Affiliations:** ^1^ Department of Obstetrics and Gynaecology Helsingborg Hospital Helsingborg Sweden; ^2^ Institution of Clinical Sciences, Department of Obstetrics and Gynaecology Lund University Lund Sweden

**Keywords:** acute pyelonephritis, asymptomatic bacteriuria, cystitis, gestational week, preterm birth, urinary tract infection

## Abstract

**Introduction:**

It is well known that urinary tract infection (UTI) during pregnancy is associated with preterm birth, but information about the risk of preterm birth depending on gestational week of infection is sparingly described in the literature.

**Material and Methods:**

An observational study based on data from Swedish registers including women giving birth 2014–2020 (*n* = 684 595). Pregnant women with UTI diagnosis were identified by the ICD‐10 codes (O230‐O239, N300, N309, and N390) from the national patient registry. Hazard ratios (HR) for preterm birth after UTI diagnosis were calculated, considering the time elapsed after infection and interactions with gestational week at UTI diagnosis. Adjustments were made for maternal age, parity, previous caesarean section, body mass index, diabetes, and smoking.

**Results:**

A diagnosis of UTI was detected in 2.8% of pregnant women. A significant association between UTI diagnosis and preterm birth was found. The aHR for preterm birth was most pronounced during the first week after a UTI diagnosis and was dependent on gestational week. The aHRs (with 95% CI) for preterm birth 0–6 days after UTI diagnosis were 18.5 (13.5–25.4), 13.5 (10.4–17.6), and 6.7 (5.6–8.0) for infections debuting week 22–27, 28–31, and 32–36, respectively. The corresponding aHRs for preterm birth 7–13 days after UTI diagnosis were 10.5 (7.2–15.2), 3.7 (2.4–5.7), and 2.9 (1.9–4.5). The increased risk for preterm birth was still significant 3 weeks or more after UTI diagnosis debuting at 22–27 weeks (aHR 2.5; 95% CI: 2.1–3.1) or at 28–31 weeks (aHR 3.2; 95% CI 2.0–4.6).

**Conclusions:**

UTI diagnosis during pregnancy is an important risk factor for preterm birth. The magnitude of the increased risk is highest 0–6 days after diagnosis; thereafter, it declines but remains significant throughout pregnancy until 37 weeks. The increased risk is especially elevated (and especially concerning) if UTI diagnosis was confirmed before 28 weeks of gestation.

## Graphical abstract

The magnitude of the increased risk for preterm birth after UTI diagnosis is dependent of the gestational week at diagnosis, with a significantly higher risk if the UTI diagnosis occurred in early‐mid pregnancy compared to episodes towards term. The increased risk was highest 0–6 days after UTI diagnosis, thereafter it declined but remained significant throughout pregnancy until term (37 weeks).

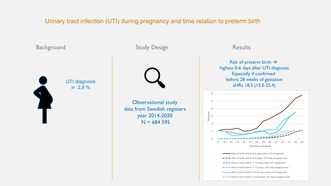

**Abbreviation**
UTI: urinary tract infection

**Key message**

A diagnosis of urinary tract infection during pregnancy was strongly associated with preterm birth. The risk was most pronounced the first week of diagnosis and before 28 weeks of gestation.

## 1 INTRODUCTION

Urinary tract infections (UTIs) are common during pregnancy with an incidence of 2%–18% and are associated with preterm birth.^1^, ^2^, ^3^ UTI includes asymptomatic bacteriuria, acute cystitis, and acute pyelonephritis.^4^ The prevalence of asymptomatic bacteriuria is similar in both pregnant and non‐pregnant women, 4%–10%,^3^, ^5^ but pregnancy increases the risk to develop acute cystitis and acute pyelonephritis.^6^

Elevated levels of progesterone cause smooth muscle relaxation, which decreases ureteric peristalsis and enhances the capacity of the bladder. This, in combination with a growing uterus compressing the ureters, generates an environment for bacterial growth.^7^

Studies regarding preterm birth after cystitis during pregnancy are limited, but acute pyelonephritis has been shown to be associated with increased maternal complications and in some studies has also been associated with preterm delivery and low birth weight.^3^ Furthermore, according to Grette et al.,^8^ there is strong evidence that acute pyelonephritis elevates the risk of preterm birth, but the scientific proof is vaguer concerning the association with acute cystitis. Asymptomatic bacteriuria solely is not regarded to increase the risk of preterm birth.^9^

Few studies have investigated the gestational week at UTI in relation to preterm birth risk. Baer et al.^10^ reported adjusted risk ratios for preterm birth, stratified for preterm birth type (<32 weeks, 32–36 weeks, and all <37 weeks), but did not consider the amount of time elapsed between the UTI and the preterm birth. To our knowledge, no such studies have been published. To fill this gap, the current study was designed to investigate the relation between the gestational week at the time of UTI diagnosis and risk of preterm birth by performing adequate time‐to‐event analyses.

## 2 MATERIAL AND METHODS

### 2.1 Data sources

Data from the Swedish nationwide antibiotics register were merged with data from the Swedish national health registers (the Swedish Medical Birth Register, MBR, and the Swedish National Patient Register, SNPR) kept by the Swedish National Board of Health and Welfare. The national 12‐digit personal identification number and date of birth were used to merge the data. The study dataset included 684 595 deliveries between 2014 and 2020.

The information in MBR is collected prospectively from standardized records at all antenatal and delivery units in Sweden. It contains high coverage (98%–99%) and high‐quality information on pregnancies and births.^11^ Information about delivery date, mode of delivery, parity, and maternal characteristics and diagnosis are available in MBR. Most pregnant women in Sweden attend the free antenatal visits offered, typically 10 visits per pregnancy. The women are interviewed by trained midwives using a standardized questionnaire. The first visit occurs at 8–12 weeks of gestation. Information about body mass index (BMI) and smoking is registered at the first antenatal visit. Smoking habits are reported by their current smoking behavior, which is categorized in the MBR as nonsmoking, 1–9 cigarettes/day, or >9 cigarettes/day. The accuracy of the self‐reported smoking habits has been validated by Mattsson et al.^12^ Maternal diagnoses are recorded as ICD‐10 codes according to the International Classification of Diseases 10th Revision (ICD‐10). The International Classification of Disease (ICD) codes for pre‐existing DM were used (E.10) for type 1 diabetes (E.11) diabetes type 2, and gestational diabetes (O.24).

The SNPR contains information about in‐ and outpatient care records, diagnoses, and admission and discharge dates from all hospitals in Sweden. The coverage is 99% and the reliability of diagnoses in the SNPR is regarded as high.^13^ The register lacks information on diagnosis given by physicians in primary care.

### 2.2 UTI diagnosis

Pregnant women with UTI were identified by the ICD‐10 codes (O230‐O239, N300, N309, and N390) from SNPR. The ICD codes are specified in Table S1. In the analysis, we only consider the first event of UTI diagnosis; those women with two or more UTI diagnoses were excluded. The reference group consists of women for whom no diagnosis of UTI during pregnancy was reported.

### 2.3 Outcomes

The outcome was preterm birth, stratified by gestational week (22–27 weeks, 28–31 weeks, 32–36 weeks).

### 2.4 Statistical methods

P‐values for groupwise comparisons between descriptive data were obtained by chi‐squared analyses; hazard ratios (HR) for preterm birth after UTI diagnosis were calculated using time‐dependent models, considering the time elapsed after infection and interactions with gestational week at UTI diagnosis. Adjustments were made for maternal age (years, continuous), primiparity (yes/no), previous CS (yes/no), BMI (kg/m^2^, continuous), gestational and pre‐existing diabetes and smoking (no, <10 cigarettes/day, ≥10 cigarettes per day, ordinal). Unknown smoking or BMI information was replaced by the overall mean. Statistical analyses were performed using IBM SPSS Statistics, version 27.0 (Armonk, NY: IBM Corp, USA).

## 3 RESULTS

The study included 684 595 deliveries in Sweden between 2014 and 2020. UTI diagnosis was detected in 2.8% of women. Table 1 shows characteristics among pregnant women with UTI diagnosis compared with the reference group (women without any diagnosis of UTI). The prevalence of women with UTI diagnosis during pregnancy was slightly (but statistically significant) higher among those with a BMI >25 kg/m^2^, height <155 cm, age <35 years, for primiparous women, and those with previous cesarean section. It was twice as common with UTI diagnosis among smoking women and those with pre‐pregnancy diabetes and 50% more frequent among women with gestational diabetes compared to women without any diagnosis of diabetes.

**TABLE 1:** Maternal characteristics by the presence of UTI diagnosis during pregnancy. Sweden 2014–2020.

	UTI during pregnancy		*p* value*
	Yes	No	
	*N* = 19 499	*N* = 665 096	
	*N* (%)	*n* (%)	
BMI (kg/m^2^)			<0.001
<25	10 417 (53.4)	372 254 (56.0)	
25–29	4.917 (25.2)	164 220 (24.7)	
30–34	2148 (11.0)	62 881 (9.5)	
35+	955 (4.9)	27 568 (4.1)	
Not known	1062 (5.4)	38 173 (5.7)	
Maternal age			<0.001
<35	15 933 (81.7)	517 665 (77.8)	
35+	3566 (18.3)	147 431 (22.2)	
Parity			<0.001
Primipara	8850 (45.4)	284 608 (42.8)	
Multipara, no previous CS	8405 (43.1)	310 422 (46.7)	
Previous CS	2244 (11.5)	70 066 (10.5)	
Smoking			<0.001
No	16 866 (86.5)	595 487 (89.5)	
Yes	1535 (7.9)	28 772 (4.3)	
Not known	1098 (5.6)	40 837 (6.1)	
Maternal height			<0.001
<155	1322 (6.8)	33 994 (5.1)	
≥155	17 842 (91.5)	614 653 (92.4)	
Not known	715 (3.7)	26 295 (4.0)	
Diabetes			
Pre‐pregnancy	298 (1.5)	4892 (0.7)	<0.001
Gestational	583 (3.0)	13 373 (2.0)	<0.001

* *p*‐value obtained from chi‐squared analysis, comparing the overall distribution, UTI cases versus reference group within each domain.

The majority (82%) of women diagnosed with UTI during pregnancy was only affected once during pregnancy. More than two episodes of UTI diagnosis were rare (*n* = 3641, 0.5%).

In the analysis, we only consider the first event of UTI diagnosis. Among the 19 499 women with UTI diagnosis during pregnancy, 1670 (8.6%) were diagnosed with pyelonephritis. The prevalence of preterm birth among women with pyelonephritis was 10.6%, similar to the overall risk of preterm birth among all women with UTI during pregnancy (10.2%).

Table 2 shows UTI diagnosis during pregnancy in relation to preterm birth. The risk of preterm birth was significantly and considerably elevated among women diagnosed with UTI compared with the reference group. The association between UTI diagnosis and preterm birth was especially evident in pregnancies affected before 28 gestational weeks. Among women diagnosed with UTI between 22 + 0 and 27 + 6 gestational weeks, 2.2% had an extreme premature birth (<28 weeks), compared to 0.3% in the reference group. The corresponding numbers in women diagnosed with UTI from 28 + 0 to 31 + 6 weeks with very preterm birth was from 2.8% to 0.5%, respectively, and among women diagnosed with UTI at 32–36 weeks, the risk for moderate to late preterm birth was 7.1% compared to 2.5% in the reference group.

**TABLE 2:** UTI diagnosis during pregnancy in relation to preterm birth.

Gestational weeks at first UTI	Women, total (*N*)	Gestational weeks at birth			
		22–27	28–31	32–36	37+
		*n* (%)	*n* (%)	*n* (%)	*n* (%)
<13 w	2461	10 (0.4)	16 (0.7)	93 (3.8)	2342 (95.2)
13–21 w	3801	32 (0.8)	39 (1.0)	167 (4.4)	3563 (93.7)
22–27 w	4744	102 (2.2)	69 (1.5)	213 (4.5)	4360 (91.9)
28–31 w	2911		82 (2.8)	174 (6.0)	2655 (91.2)
32–36 w	3303			236 (7.1)	3067 (92.9)
37 + w	2279				2279 (100)
No UTI	665 096	2023 (0.3)	3527 (0.5)	16 347 (2.5)	643 199 (96.7)

**FIGURE 1:**
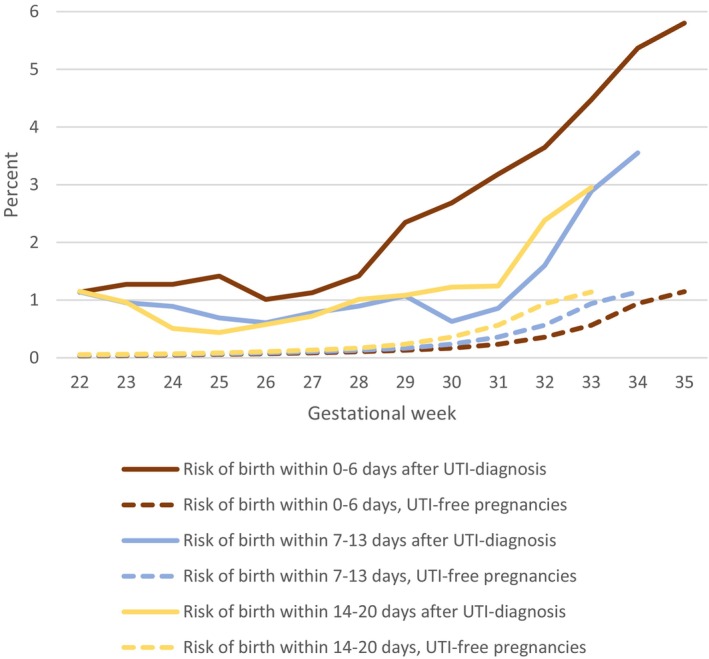
UTI diagnosis and risk for preterm birth within 1, 2 or 3 weeks after diagnosis compared to the risk of preterm birth among all ongoing UTI free pregnancies (reference group).

Figure 1 shows the risk for preterm birth within 1, 2, or 3 weeks after UTI diagnosis by gestational week at diagnosis and the corresponding risk among all UTI‐free ongoing pregnancies at each specified gestational week, respectively. Irrespective of gestational week, the increased risk was highest 0–6 days after UTI diagnosis, but the increased risk for preterm birth was still evident 7–13 or 14–20 days after UTI diagnosis.

Table 3 shows the crude HR and adjusted HR for extreme, very, and moderate preterm birth. Compared to the reference group, the increased risk for preterm birth was highest 0–6 days after UTI diagnosis, and especially high for UTI diagnosis in gestational week 22–27 + 6 (aHR 18.5; 95% CI 13.5–25.4) or 28–31 + 6 weeks (aHR 13.5; 95% CI 10.4–17.6), respectively. For UTI diagnosis at 32–36 gestational weeks, the corresponding aHR was 6.7 (95% CI 5.6–8.0). Adjustments for maternal age, parity, smoking, gestational and preexisting diabetes, and BMI affected the estimates marginally.

**TABLE 3:** Crude and adjusted hazard ratio for extreme, very, and moderate preterm birth, respectively, by time elapsed from UTI diagnosis.

Interval between UTI and preterm birth	Gestational age at UTI											
	22–27 weeks				28–31 weeks				32–36 weeks			
	HR	95% CI	HR^a^	95% CI	HR	95% CI	HR^a^	95% CI	HR	95% CI	HR^a^	95% CI
0–6 days	19.1	14.0–26.3	18.5	13.5–25.4	13.5	10.4–17.6	13.3	10.3–17.4	6.8	5.7–8.2	6.7	5.6–8.0
7–13 days	10.8	7.4–15.7	10.5	7.2–15.2	3.7	2.4–5.7	3.7	2.4–5.7	3.0	2.0–4.6	2.9	1.9–4.5
14–20 days	7.8	5.3–11.5	7.5	5.1–11.2	3.4	2.2–5.2	3.3	2.2–5.1	—	—	—	—
≥21 days	2.6	2	2.5	2.1–3.1	3.2	2.1–4.8	3.0	2.0–4.6	—	—	—	—
UTI‐free ongoing pregnancies	1.0	Reference	1.0	Reference	1.0	Reference	1.0	Reference	1.0	Reference	1.0	Reference

^a^ Adjusted for maternal age, parity, previous CS, BMI, smoking, gestational and pregestational diabetes.

**FIGURE 2:**
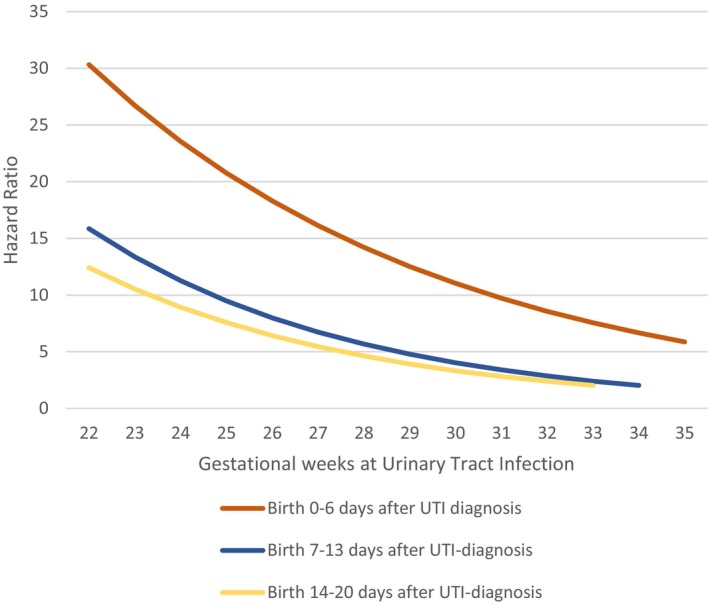
Hazard ratio for preterm birth within 1, 2, or 3 weeks, by timing of UTI diagnosis, considering the interaction between the increased risk for preterm birth and gestational week at UTI.

Figure 2 shows the HR for birth within 1, 2 or 3 weeks, by timing of UTI diagnosis, considering the interaction between increased risk for preterm birth and gestational week of diagnosis. The HR for preterm birth was highest 0–6 days after UTI diagnosis and decreased with advancing gestational week (from 30.3 at 22 + 0 weeks to 5.9 at 35 + 6 weeks). The corresponding HRs 7–13 days after UTI diagnosis were 15.9 and 2.0 at 34 + 0 weeks, respectively.

## 4 DISCUSSION

We confirmed previous studies identifying UTI during pregnancy as an important risk factor for preterm birth. Above all, we found a significant interaction between gestational week at diagnosis and increased risk. The increased risk was especially elevated and concerning if UTI was diagnosed before 28 weeks of gestation. Irrespective of gestational week of UTI diagnosis, the magnitude of the increased risk was highest 0–6 days after diagnosis; thereafter, it declined, but the increased risk for preterm birth remained significant throughout pregnancy until 37 weeks. This strong association might be explained by infection causing an inflammatory response, which results in contractions of the uterus and can lead to preterm birth.^14^, ^15^ The findings may have important clinical implications. Being diagnosed with UTI, especially if diagnosed before 28 gestational weeks, implies a risk of preterm birth. This information is valuable for both clinicians and patients.

The incidence of UTI in our study was 2.8% compared with an estimated incidence of 2%–15% in a previous narrative meta‐analysis.^3^ The reason for the broad span of prevalence is likely caused by the heterogeneity of the conditions that are gathered under the UTI umbrella. Some studies exclusively include cystitis and acute pyelonephritis while others also include asymptomatic bacteriuria. An RCT published in Lancet 2015^9^ found no association between preterm birth and asymptomatic bacteriuria in uncomplicated singleton pregnancies. We did not include asymptomatic bacteriuria in the current study.

The issue of the timing of UTI in relation to preterm birth risk was, to some extent, addressed in the study of Baer et al.^10^ The investigators explored the increased risk for preterm birth, stratified into <32 weeks and 32–36 weeks, by trimester at UTI diagnosis. Compared to women without UTI, the risk ratio for preterm birth <32 weeks was somewhat higher than the corresponding risk ratio for preterm birth 32–36 weeks (1.4 and 1.1, respectively) among women diagnosed with UTI during the first trimester. Based on these results, the authors concluded that the association between UTI and preterm birth was present regardless of trimester of pregnancy. However, in the quoted study, the time to event was not considered, and no interaction between gestational week at diagnosis and preterm birth risk was formally tested for.

The strength of the current study is that it is based on a large, population‐wide, high‐quality Swedish national registers, which limits bias. Furthermore, the large cohort used in the current study enabled detailed analysis on the time elapsed from UTI diagnosis to preterm birth. Limitations include the risk of confounding and reversed causality. The risk of reversed causality is present since women seeking care for premature contractions may be likely to be screened for bacteriuria and diagnosed with UTI.

Furthermore, there is a risk of both over‐ and underestimating the frequency of UTI diagnosis. There is a risk of overestimating UTI frequency since the threshold for treating a suspected UTI is low, due to the awareness of the elevated risk of UTI and preterm birth in pregnancy. On the other hand, SNPR lacks information from primary care which inevitably results in an underestimating of UTI frequency. It can be assumed that the cases included in the study are more severe than those that are not identified, since primary care often treats less severe conditions.

Despite the limitations regarding diagnosis reliability, the awareness of the association between UTI diagnosis and preterm birth could be used to identify women with increased risk for preterm birth.

The benefits of treatment of asymptomatic bacteriuria to prevent preterm birth are debated. Kazemier et al.^9^ concluded that asymptomatic bacteriuria was not associated with preterm birth among women with an uncomplicated singleton pregnancy. However, a systematic review from 2019,^16^ mostly based on older studies, found evidence for a slightly protective effect of antibacterial treatment of asymptomatic bacteriuria on the risk of preterm birth. Regarding the benefits of adequate treatment for symptomatic UTI, there are no available results from randomized trials. We assume that all women who were diagnosed with symptomatic UTI in our study retrieved antibacterial treatment. Swedish guidelines recommend testing for asymptomatic bacteriuria by urine culture only for women with risk factors (previous acute pyelonephritis, repeated urinary tract infections (>3), urinary tract infection during previous pregnancy, diabetes, chronic kidney disease, autoimmune disease with kidney engagements, and for those with genital mutilation). After an established UTI, it is recommended to follow up with a urine culture 1–2 weeks after completed antibiotic course. The result in the current study demonstrates that women diagnosed with UTI have a distinct risk of preterm birth compared to the reference group.

Our results highlight the importance of raising awareness of the risk of preterm birth among women and healthcare personnel as well as the importance of carefully adhering to the national guidelines.

## 5 CONCLUSION

The magnitude of the increased risk for preterm birth after UTI diagnosis is dependent on the gestational week at diagnosis, with a significantly higher risk if the UTI diagnosis occurred in early‐mid pregnancy compared to episodes toward term. The increased risk was highest 0–6 days after UTI diagnosis; thereafter, it declined but remained significant throughout pregnancy until term (37 weeks). Our findings imply the importance of recognizing these women as a risk group for preterm birth.

## AUTHOR CONTRIBUTIONS

KD, AS, and KK designed, carried out, and wrote the paper. KK performed the statistical analysis; all authors were present and analyzed the data. All authors approved the final version of the manuscript.

## CONFLICT OF INTEREST STATEMENT

None.

## ETHICS STATEMENT

This research was conducted in accordance with the principles embodied in the Declaration of Helsinki with local statutory requirements. Ethical Approval was given on August 2, 2018 (Dnr 2018/539) by the Committee of ethics at Lund University.

## FUNDING STATEMENT

The Gorthon Foundation for Medical Research provided funds and regional support for research time (Karin Dahlquist). The funders had no role in conducting the study or in the writing of the paper.

## Supporting information


**Table S1.** ICD‐10 codes used for urinary tract infection collected from the national patient registry.
**Table S2.** Hazard ratios for premature birth by the presence of UTI diagnosis, interval between UTI diagnosis and preterm birth, and maternal characteristics and diseases. Results from multivariable Cox regression analyses.

## Data Availability

The data that support the findings of this study are openly available in [Acta Obstetetric and Gynecology Scandinavia] at [DOI:10.1111/aogs.70156], reference number [2026;00 : 1‐7].
