# Supplementary material for: Urinary tract infection during pregnancy and time relation to preterm birth—a Swedish observational study
Source: Acta Obstet Gynecol Scand. 2026 Feb 13;105(4):672–8. doi: 10.1111/aogs.70156 (PMC13140709; doi:10.1111/aogs.70156)
Supplement: Supplementary file 1 — Table S1. ICD‐10 codes used for urinary tract infection collected from the national patient registry. Table S2. Hazard ratios for premature birth by the presence of UTI diagnosis, interval between UTI diagnosis and preterm birth, and maternal characteristics and diseases. Results from multivariable Cox regression analyses. [file AOGS-105-672-s001.docx]

S1

ICD-10 codes used for urinary tract infection collected from the national patient registry

O23.0 Infection in the kidney during pregnancy

O23.1 Cystitis during pregnancy

O23.2 Urethritis during pregnancy

O23.3 Infections in other parts of the urinary organ during pregnancy

O23.4 Unspecified infection in the urinary organ during pregnancy

O23.5 Infections in the reproductive organ during pregnancy

O23.9 Other an unspecified infection in the urinary and reproductive organ during pregnancy

N30.0 Acute cystitis

N30.9 Cystitis, unspecified

Table S2. Hazard Ratios for premature birth by presence of UTI diagnosis, interval between UTI diagnosis and preterm birth, and maternal characteristics and diseases. Results from multivariable Cox regression analyses.

|  |  | Adjusted Hazard Ratio | | |  |
| --- | --- | --- | --- | --- | --- |
|  |  | aHR | 95%CI | | p-value |
| **UTI at 22-27 weeks** | |  |  |  |  |
|  | Interval between UTI and preterm birth:  0-6 days | 18.51 | 13.49 | 25.40 | 0.000 |
|  | 7-13 days | 10.45 | 7.21 | 15.17 | 0.000 |
|  | 14-20 days | 7.53 | 5.08 | 11.16 | 0.000 |
|  | >=21 days | 2.53 | 2.06 | 3.11 | 0.000 |
|  | BMI  (per one unit increase) | 1.01 | 1.01 | 1.01 | 0.000 |
|  | Smoking  (per one unit increase) | 1.39 | 1.34 | 1.43 | 0.000 |
|  | Age  (per one year increase) | 1.02 | 1.02 | 1.02 | 0.000 |
|  | Pre-gestational Diabetes | 4.07 | 3.83 | 4.32 | 0.000 |
|  | Gestational diabetes | 1.35 | 1.28 | 1.44 | 0.000 |
|  | Primipara | 1.73 | 1.69 | 1.77 | 0.000 |
|  | Previous CS | 1.43 | 1.39 | 1.48 | 0.000 |
|  |  |  |  |  |  |
| **UTI at 28-31 weeks** | |  |  |  |  |
|  | Interval between UTI and preterm birth:  0-6 days | 13.34 | 10.25 | 17.36 | 0.000 |
|  | 7-13 days | 3.68 | 2.40 | 5.65 | 0.000 |
|  | 14-20 days | 3.32 | 2.16 | 5.10 | 0.000 |
|  | >=21 days | 3.03 | 1.99 | 4.61 | 0.000 |
|  | BMI  (per one unit increase) | 1.01 | 1.01 | 1.02 | 0.000 |
|  | Smoking  (per one unit increase) | 1.39 | 1.35 | 1.44 | 0.000 |
|  | Age  (per one year increase) | 1.02 | 1.02 | 1.02 | 0.000 |
|  | Pre-gestational Diabetes | 4.13 | 3.89 | 4.39 | 0.000 |
|  | Gestational diabetes | 1.34 | 1.27 | 1.43 | 0.000 |
|  | Primipara | 1.73 | 1.69 | 1.77 | 0.000 |
|  | Previous CS | 1.43 | 1.38 | 1.48 | 0.000 |
| **UTI at 32-36 weeks** | |  |  |  |  |
|  | Interval between UTI and preterm birth:  0-6 days | 6.73 | 5.64 | 8.03 | 0.000 |
|  | 7-13 days | 2.94 | 1.93 | 4.46 | 0.000 |
|  | BMI  (per one unit increase) | 1.01 | 1.01 | 1.02 | 0.000 |
|  | Smoking  (per one unit increase) | 1.39 | 1.34 | 1.43 | 0.000 |
|  | Age  (per one year increase) | 1.02 | 1.02 | 1.02 | 0.000 |
|  | Pre-gestational Diabetes | 4.10 | 3.86 | 4.35 | 0.000 |
|  | Gestational diabetes | 1.34 | 1.27 | 1.43 | 0.000 |
|  | Primipara | 1.73 | 1.69 | 1.77 | 0.000 |
|  | Previous CS | 1.43 | 1.38 | 1.48 | 0.000 |
